# Neuroprotection Afforded by an Enriched Mediterranean-like Diet Is Modified by Exercise in a Rat Male Model of Cerebral Ischemia

**DOI:** 10.3390/antiox13020138

**Published:** 2024-01-23

**Authors:** Daniel Romaus-Sanjurjo, María Castañón-Apilánez, Esteban López-Arias, Antía Custodia, Cristina Martin-Martín, Alberto Ouro, Elena López-Cancio, Tomás Sobrino

**Affiliations:** 1 NeuroAging Group (NEURAL), Clinical Neurosciences Research Laboratory (LINC), Health Research Institute of Santiago de Compostela (IDIS), 15706 Santiago de Compostela, Spain; daniel.romaus.sanjurjo@sergas.es (D.R.-S.); esteban.lopez.arias@sergas.es (E.L.-A.); antia.custodia.malvido@sergas.es (A.C.); tomas.sobrino.moreiras@sergas.es (T.S.); 2 Centro de Investigación Biomédica en Red en Enfermedades Neurodegenerativas, Instituto de Salud Carlos III, 28029 Madrid, Spain; 3 Departament of Neurology, Hospital Universitario Central de Asturias (HUCA), 33011 Oviedo, Spain; maria.castanon@sespa.es; 4 Instituto de Investigación Sanitaria del Principado de Asturias (ISPA), 33011 Oviedo, Spain; 5 Department of Functional Biology, Universidad de Oviedo, 33003 Oviedo, Spain; 6 Translational Immmunology, Instituto de Investigación Sanitaria del Principado de Asturias (ISPA), 33011 Oviedo, Spain; cmartinsorting@finba.es

**Keywords:** cerebral ischemia, EPCs, exercise, hydroxytyrosol, inflammation, mediterranean diet, microglia, Milliplex, neuroprotection, tMCAO

## Abstract

Ischemic stroke is an important cause of mortality and disability worldwide. Given that current treatments do not allow a remarkably better outcome in patients after stroke, it is mandatory to seek new approaches to preventing stroke and/or complementing the current treatments or ameliorating the ischemic insult. Multiple preclinical and clinical studies highlighted the potential beneficial roles of exercise and a Mediterranean diet following a stroke. Here, we investigated the effects of a pre-stroke Mediterranean-like diet supplemented with hydroxytyrosol and with/without physical exercise on male rats undergoing transient middle cerebral artery occlusion (tMCAO). We also assessed a potential synergistic effect with physical exercise. Our findings indicated that the diet reduced infarct and edema volumes, modulated acute immune response by altering cytokine and chemokine levels, decreased oxidative stress, and improved acute functional recovery post-ischemic injury. Interestingly, while physical exercise alone improved certain outcomes compared to control animals, it did not enhance, and in some aspects even impaired, the positive effects of the Mediterranean-like diet in the short term. Overall, these data provide the first preclinical evidence that a preemptive enriched Mediterranean diet modulates cytokines/chemokines levels downwards which eventually has an important role during the acute phase following ischemic damage, likely mediating neuroprotection.

## 1. Introduction

Ischemic stroke remains one of the leading causes of mortality and disability worldwide [1]. Following the occlusion of a blood vessel, the pathological paths of the ischemic cascade are activated, which eventually leads to neuronal death by necrosis or apoptosis [2]. Unfortunately, although the current reperfusion therapies (mechanical thrombectomy and thrombolysis) are currently extended to larger therapeutic windows and larger infarcts, still less than one-third of patients receive those treatments [3,4,5], and the probability of a long-term good functional outcome is still lower than 50% even after a successful reperfusion treatment. Therefore, new approaches such as stroke-preventing alternatives and/or complementary treatments to reperfusion therapies are needed to improve the recovery of stroke patients. Along these lines, regular exercise and a Mediterranean diet are associated with lower rates of cardiovascular and cerebrovascular disease [6,7,8]. Importantly, beyond the impact on suffering from stroke, both exercise and diet may have positive effects on neuroprotection and neurorepair mechanisms following ischemia [9,10]. 

Exercise aids stroke recovery through processes such as vasculogenesis and neurogenesis; however, the exact molecular mechanisms are unclear [11]. Several studies showed that pre-stroke physical activity was associated with a lower initial severity and infarct volume as well as a better short-term functional outcome [12,13,14]. Furthermore, clinical studies from our group revealed that a higher rate of pre-stroke exercise reduced the severity and the infarct volume and increased the rate of recanalization following recombinant tissue plasminogen activator (rtPA) treatment and long-term functional recovery. This beneficial effect might be mediated by an increase in vascular endothelial growth factor (VEGF) levels [15,16].

The Mediterranean diet is based on the abundant consumption of food such as fruits, vegetables, legumes, nuts, and olive oil as the principal fat source, among others. It provides several beneficial nutrients (e.g., monounsaturated fatty acids), a well-balanced proportion of the essential fatty acids omega-3/omega-6, and high amounts of fiber and antioxidants [17,18]. So far, numerous in vivo and in vitro studies have highlighted the importance of polyphenols as neurorepair substances following ischemia through anti-apoptotic, antioxidant, and anti-inflammatory mechanisms due to their ability to cross the blood-brain barrier (BBB) [19,20,21,22,23,24,25]. Specifically, hydroxytyrosol (HT), an extra-virgin olive oil-derived polyphenol, has been suggested as a promising therapeutic compound following a stroke due to its biological effects such as antioxidant, anti-inflammation, and neuroprotection [26,27,28,29,30,31]. Preclinical studies have shown that the pre-stroke consumption of either olive oil or olive leaf extract positively impacts cellular and behavioral outcomes following ischemia [28,29]. Similarly, post-stroke administration of HT also revealed beneficial effects in the recovery of animals undergoing a stroke [30,31]. Results from clinical studies assessing the relationship between a Mediterranean diet and stroke have observed associations with less atherosclerotic etiology and better outcomes, specifically with the consumption of olive oil [32,33,34,35,36].

Physical activity and a balanced Mediterranean diet may exert synergistic effects on ischemic tolerance. Both the Mediterranean diet and exercise have been also postulated as endothelial progenitor cell (EPC)-stimulating factors, which mediate vasculogenesis and the optimization of endothelial function following a stroke [37,38]. EPCs are a subpopulation of CD34+ bone marrow-derived progenitor cells (BMPCs) that exhibit characteristics of both endothelial and stem cells, participating in angiogenesis and/or the maintenance of the endothelium [39]. Importantly, our group previously demonstrated the positive role of EPCs within the first week following a stroke [40,41,42]. Moreover, some previous studies evaluated the synergy of exercise and diet in cognition [43,44,45,46]; nevertheless, to our knowledge, no studies have assessed the potential synergistic effect of combining both exercise and diet previously to stroke.

Our study investigated the combined effect of pre-stroke exercise and a Mediterranean diet on reducing infarct size and improving post-stroke recovery.

## 2. Materials and Methods

### 2.1. Animal Handling

Male adult Sprague-Dawley (SD) rats (300–350 g, 9–10 weeks of age) were used. Two rats per cage were kept with day/night cycles of 12/12 h at a mean temperature of 22 ± 1 °C and humidity of 60 ± 5%, and they had water and food ad libitum. Housing conditions obeyed the Spanish legislation for experimental animals (Real Decreto 53/2013). For surgical procedures and magnetic resonance imaging (MRI) acquisitions, anesthesia was induced by inhalation of 6% sevoflurane in a N_2_O/O_2_ mixture (70/30) and maintained at 3–4% sevoflurane during procedures. Body temperature was maintained at 37 °C with a feedback-controlled heating pad (Neos Biotec, Pamplona, Spain) until animals completely recovered from anesthesia.

**Figure 1:**
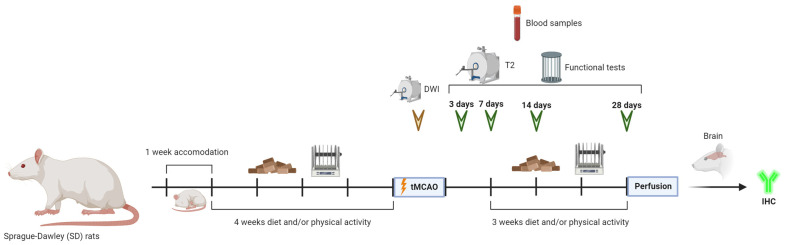
Experimental protocol. Schematic drawing of the protocols followed throughout the time course of the experiments.

Forty-five SD rats were randomly divided for each condition as follows: 5 rats for the sham group, 10 rats for the control group (normal diet and no physical activity), 10 rats for the diet group (Mediterranean-like rich in polyphenols diet and no physical activity), 10 rats for the PA group (normal diet and physical activity) and 10 rats for the diet/PA group (Mediterranean-like rich in polyphenols diet and physical activity) (Figure 1).

### 2.2. Physical Activity

Animals from PA and diet/PA groups performed physical activity sessions (rotarod) five days per week during the 4 weeks prior to surgery consisting of 5 min accelerating from 0.5 to 3 m/min plus 20 min at 3 m/min. All animals were previously adapted to rotarod during the first week by performing 5 min at 0.5 m/min. Following surgery, animals were allowed to recover for one week and then they performed physical activity for 3 more weeks until the final follow-up. Control and diet rats performed control training sessions consisting of the placement of the animal in stopped rotarod. 

### 2.3. Diet

Within the first week in the animal facility, and during the resting week following surgery, all rats were fed the same food (Teklad Global 19% Protein Extruded Rodent Diet, ref: 2018, ENVIGO, Indianapolis, IN, USA). Then, rats from the sham, control, and PA groups were fed a Western-like diet (control diet with dairy butter as the fat source, ref: U8978-version-0177, AIN93, SAFE, Augy, France) during the 4 weeks prior to surgery. Animals from the diet and diet/PA groups were fed with a Mediterranean-like diet containing 85% olive oil/15% anchovy sardine oil as a fat source (ref: U8978 Version 01 AIN93, SAFE, France) during the 4 weeks prior to surgery and for 3 more weeks until the final follow-up. Moreover, rats from the diet and diet/PA groups were supplied with a supplement of HT (ref.: 21001, Nutexa, Ontinyent, Spain) diluted in the water (200 mg/kg/day). In all cases, the food was available ad libitum. 

### 2.4. Surgical Procedures

Transient focal ischemia was induced in rats by using the transient middle cerebral artery occlusion (tMCAO) model, which is considered one of the best models to mimic human ischemic stroke and has been used in numerous studies [47,48,49]. The tMCAO surgery was performed as previously described [47] with slight modifications. Briefly, the left common, external, and internal carotid arteries were dissected from connective tissue through a midline neck incision under a surgical microscope (Leica MZ6, Leica Microsystems, Wetzlar, Germany). Then, the left external carotid artery and pterygopalatine artery of the internal carotid artery were separated and ligated by 6-0 silk sutures. A silicon rubber-coated monofilament (403512PK5Re; Doccol Corporation, Sharon, MA, USA) was inserted through the external carotid into the left common carotid artery and advanced into the internal carotid artery to 20 mm from the bifurcation to occlude the origin of the MCA. A laser-Doppler flow probe (tip diameter 1 mm) attached to a flowmeter (PeriFlux 5000, Perimed AB, Järfälla, Sweden) was located over the thinned skull in the MCAO territory (4 mm lateral and 1 mm posterior to bregma) to obtain a continuous measurement of relative cerebral brain flow during the arterial occlusion. Once the artery occlusion was achieved, as indicated by Doppler signal reduction, each animal was carefully moved from the surgical bench to the magnetic resonance imaging (MRI) system for ischemic lesion assessment using apparent diffusion coefficient maps (defined as T0 ischemic lesion). After magnetic resonance (MR) analysis, the animals were returned to the surgical bench and the Doppler probe was repositioned. The suture was removed after 75 min of occlusion and the left pterygopalatine artery was reperfused while the left external carotid (used to introduce the suture) remained tied to avoid bleeding. 

### 2.5. Animal Experimental Procedures

The following exclusion criteria were used: (1) a less than 70% reduction in relative cerebral blood flow; (2) arterial malformations, as determined by MRA; (3) a baseline lesion volume of less than 25% or greater than 45% with respect to the ipsilateral hemisphere, as measured using ADC maps; and (4) the absence of reperfusion or prolonged reperfusion (more than 10 min until the achievement of at least 50% of the baseline cerebral blood flow) after monofilament removal. As previously described [50], this surgical protocol represents a reliable method to reduce intergroup variability and to guarantee the reproducibility of the infarct volumes during the tMCAO surgery. 

Experimental procedures were performed following five criteria derived from the Stroke Therapy Academic Industry Roundtable (STAIR) group guidelines for preclinical evaluation of stroke therapeutics [51,52]: (1) the cerebral blood flow was measured to confirm the vascular occlusion as an index of the reliability of the ischemic model; (2) the animals were randomly assigned to treatment groups of the study; (3) the researchers were blinded to the treatment administration; (4) the researchers were blinded to the treatments during outcome assessment; and (5) the temperature was monitored during the surgery.

### 2.6. Magnetic Resonance Image Protocol

The MRI studies were conducted on a 9.4-T horizontal bore magnet MRI system, Biospec 94/20USR (Bruker BioSpin, Bremen, Germany), with 20 cm wide actively shielded gradient coils (440 mT/m). Radiofrequency transmission was achieved with a birdcage volume resonator. The signal was detected using a four-element surface coil positioned over the head of the animal, which was fixed with a tooth bar, earplugs, and adhesive tape. Transmission and reception coils were actively decoupled from each other.

Gradient-echo pilot scans were performed at the beginning of each imaging session for the accurate positioning of the animal inside the magnet bore. The axial magnetic resonance angiography (MRA) images of the rat brain were a stack of 58 slices mapping the whole brain. The parameters for the TOF-MRA 3D Flash sequence were a field-of-view (FOV) of 30.72 × 30.72 × 14 mm^3^, an image matrix of 256 × 256 × 58, a repetition time of 15 ms with 2 averages, and an echo time of 2.5 ms. Immediately after the MRA, diffusion coefficient maps and ADC maps were acquired using a spin-echo echo-planar imaging sequence with the following acquisition parameters: an FOV of 24 × 16 mm^2^, an image matrix of 96 × 64, 14 consecutive slices of 1 mm thickness, a repetition time of 4 s with 4 averages, an echo time of 26.91 ms, a spectral bandwidth of 200.000 Hz, and 7 diffusion b values: 0, 300, 600, 900, 1200, 1600, and 2000. T2-weighted images were acquired at 3, 7, 14, and 28 days after tMCAO using a RARE (factor *n* = 4) sequence with the following acquisition parameters: an FOV of 19.2 × 19.2 mm^2^, an image matrix of 192 × 192, 14 consecutive slices of 1 mm thickness, a repetition time of 3 s, and an effective echo time of 45 ms.

MRA imaging was performed to ensure that the artery remained occluded throughout the procedure in a non-invasive manner. Moreover, a basal ischemic lesion during tMCAO was determined by counting the pixels with ADC values below a threshold in the ipsilateral brain hemisphere. The values of ADC in the healthy rat brain normally do not fall below 0.55 × 10^−3^ mm^2^/s; therefore, this threshold provided a convenient means of segmenting abnormal tissue [50]. The DWI and MRA images were simultaneously acquired during tMCAO. 

All images were processed, and the maps were constructed with ImageJ software (https://imagej.net/ij/, accessed on 18 January 2024). Diffusion volumes were determined from diffusion-weighted image (DWI) maps, whereas infarct volumes were determined from T2 maps, both by manual selection. Edema was estimated by measuring the volumes of the affected (VLes) and contralateral (Vc) hemispheres and using the formula:

edema (%) = 100 × [(VLes − Vc)/Vc].

The Vc/VLes ratio was also used to correct lesion volumes for edema formation.

### 2.7. Functional Tests

All animals underwent a battery of functional tests in order to assess their motor function (cylinder test) and evaluate their sensorimotor deficit (Bederson and Wahl’s tests) at different time points during the darkness cycle: baseline (before tMCAO) and 3, 7, 14, and 28 days following surgery. 

A cylinder test was performed to evaluate limb asymmetry during the exploratory activity, and therefore, the spontaneous use of forelimbs, as previously described [53]. Briefly, each animal was put in a cylinder with a 20 cm diameter transparent base and a video camera underneath recording the vertical exploratory movements of animals. Then, the laterality index was calculated as the number of times that the animal touched the cylinder with the right leg during the ascendant movement divided by the number of times that the animal touched with each leg. This index is close to 0.5 for healthy animals and tends to be 0 or 1 for animals that have preferential use of the left or the right paw, respectively [54].

On the other hand, sensorimotor deficits were evaluated with both Bederson and Wahl scales that mainly assess the presence or absence of reflex and spontaneous activity of the animal [55,56]. The Bederson scale included the following items: spontaneous movement, spontaneous rotation, spontaneous flexion of the contralateral forelimb, edge detection, turn after tail suspension, and reflection of protection. Likewise, the Wahl scale included the following items: edge detection, visual placing, spontaneous flexion of contralateral forelimb and hindlimb, circular spontaneous movement, and both extensor reflex and thoracic torsion when suspended.

### 2.8. Flow Cytometry Analysis of Hematopoietic Lineages 

Blood samples (0.5 mL) were drawn from the tail vein before tMCAO (basal sample) and at 1 h, and at 3, 7, 14, and 28 days after tMCAO. The samples were collected into K2EDTA tubes (BD Microtainer, Franklin Lakes, NJ, USA). Immunofluorescence cell staining was performed with the fluorescent conjugated antibodies anti-CD34 (ref.: sc-7324, Santa Cruz Biotechnology, Dallas, TX, USA), anti-ckit (ref.: sc-19619, Santa Cruz Biotechnology, Dallas, TX, USA), anti-KDR (ref.: bs-10412R, Bioss, Woburn, MA, USA), and anti-CD43 (ref.: 202814, Biolegend, San Diego, CA, USA). Cell fluorescence was measured 15 min after staining by flow cytometry with BD FACS Aria II (BD, Bioscience, Franklin Lakes, NJ, USA). The numbers of each cellular lineage were calculated using the FACSDiva software (https://www.bdbiosciences.com/en-us/products/software/instrument-software/bd-facsdiva-software, accessed on 18 January 2024; BD Biosciences, Franklin Lakes, NJ, USA).

### 2.9. Immunomodulator Effect and Oxidative DNA Damage

Blood samples were drawn from the tail vein before tMCAO (basal sample) and at 1 h, and at 3, 7, 14, and 28 days after tMCAO. The samples were collected into K2EDTA tubes (BD Microtainer, USA) and centrifugated at 1000× *g* for 7 min, storing serum at −80 °C until analysis. Serum levels of cytokines/chemokines were determined using a Milliplex MAP Rat Cytokine/Chemokine Magnetic Bead Panel kit (Cat. RECYTMAG-65K, EMD Millipore, Darmstadt, Germany). Similarly, the measurement of oxidative DNA damage in serum was performed with an ELISA kit (8-hydroxydeoxyguanosine assay, 8-OHdG) (Cat. STA-320; Cell Biolabs, San Diego, CA, USA).

### 2.10. Tissue Processing

Four animals per group at 14 days, and the rest of them at 28 days, were sacrificed by an overdose of anesthesia, and then transcardially perfused with an ice-cold solution of PBS followed by 4% paraformaldehyde (PFA). Finally, the brains were dissected out, and the tissues were post-fixed overnight at 4 °C in the same fixative solution.

Tissue processing was performed as described previously [57]. Briefly, tissues were incubated in 30% sucrose for cryo-protection. The brains were embedded in an OCT compound and frozen. The tissues were transversely sectioned with a cryostat at a thickness of 20 μm. For immunohistochemistry, the sections were first washed with 0.2% triton-PBS, and the antigen retrieval protocol was carried out with sodium citrate (pH 6.0) at 99 °C for 20 min. Then, the sections were blocked and permeabilized (0.4% triton and 5% sera matching the species of the secondary antibodies, 1× PBS) for 1 h at room temperature. Incubation in the primary antibody solution (see concentrations below) was carried out at 4 °C overnight. The sections were washed, followed by secondary antibody staining for 2 h at room temperature (antibody solutions at 1:500). Next, the sections were washed again, incubated with Hoechst 33342 for 10 min, and mounted with Aqua-Poly/Mount (Polysciences, Warrington, PA, USA).

The following antibodies were used: mouse anti-Ki67 (ref.: M724029-2, 1:50, Agilent Technologies, Santa Clara, CA, USA), rabbit anti-DCX (ref.: ab18723, 1:500, Abcam, Cambridge, UK), guinea pig anti-NeuN (ref.: ABN90, 1:200, Sigma-Aldrich, Burlington, MA, USA), goat anti-CD31 (ref.: AF3628, 1:100, Novus Biologicals, Abingdon, UK), rabbit anti-VGLUT1 (ref.: 12331, 1:100, Cell Signaling Technology, Danvers, MA, USA), rabbit anti-Iba1 (ref.: MA5-36257, 1:100, Thermo Scientific, Waltham, MA, USA), and anti-IB4 (ref.: L2140, 1:50, Sigma-Aldrich).

### 2.11. TUNEL Labeling

The Tdt-mediated dUTP Nick End Labelling (TUNEL) Kit (Roche, Mannheim, Germany) was used to detect apoptotic nuclei as previously described [58,59]. Briefly, sections were pre-treated with MetOH at −20 °C for 15 min and then with 0.01 M of citrate buffer at a pH of 6.0 for 30 min at 90 °C. Following the washes with PBS, each slide was incubated with a mixture of 5 μL of enzyme solution (terminal deoxynucleotidyl transferase) and 45 μL of labeling solution (TMR red labeled nucleotides) for 90 min at 37 °C. Then, the sections were rinsed in PBS, allowed to dry for 30 min at 37 °C, and mounted with Aqua-Poly/Mount.

### 2.12. Image Acquisition

Stained tissue sections were photographed using an inverted microscope (Leica CTR6000, Leica Microsystems, Wetzlar, Germany). Three sections from coronal brain sections were photographed for each animal. Photomicrographs were taken at 5× (areas DCX+ or CD31+) and 20× (TUNEL, neurogenesis, angiogenesis, synapsis, and microglia studies) magnification without changing the amplifier gain or the offset to avoid the introduction of experimental variability. All images were processed with ImageJ software (https://imagej.net/ij/, accessed on 18 January 2024).

Following quantifications, the contrast and brightness were minimally and uniformly adjusted in the figures, across panels for each experiment, with Adobe Photoshop CS4 (Adobe Systems, San Jose, CA, USA).

### 2.13. Immunofluorescence Quantifications

To quantify the intensity of each immunofluorescence (IF) signal in the cortical area surrounding the ischemic area (Iba1, IB4, and vGLUT1 signals), mean fluorescent intensity (mean grey value) was determined using ImageJ software (https://imagej.net/ij/, accessed on 18 January 2024). All values underwent internal normalization to the same contralateral areas and then they were normalized against control values. The number of TUNEL+ neurons was calculated by quantifying the NeuN+/TUNEL+ cells in the cortical area surrounding the ischemic area, in the same way for the numbers of Iba1+ cells. The experimenter was blinded during quantifications.

For neurogenesis analysis, Ki67+ cells were measured within the DCX+ area at the subventricular zone (SVZ). Similarly, angiogenesis was calculated by measuring the number of Ki67+ cells within CD31+ vessels present at the SVZ.

### 2.14. Statistical Analysis

The sample size was calculated using EPIDAT software 4.2 (http://www.sergas.es/Saude-publica/EPIDAT-4-2, accessed on 18 January 2024), based upon α  =  0.05 and the power of β  =  0.8. Statistical analysis was carried out using Prism 8 (GraphPad Software, version 8.0.1, La Jolla, CA, USA). Data were presented as the mean  ±  S.E.M. Normality of the data was determined by two different tests depending on the *n* numbers: the D’Agostino-Pearson omnibus test when *n* numbers were equal or higher than 10, and the Shapiro–Wilk normality test when *n* numbers were below 10. Data with multiple comparisons were analyzed by either a one-way ANOVA or Kruskal–Wallis test where appropriate, and post-hoc Dunn’s multiple comparisons tests. Correlation analysis was assessed with the Spearman correlation coefficient test. The significance level was set at 0.05. In the figures, significance values were represented by different numbers of asterisks: * *p*  <  0.05; ** *p*  <  0.01; *** *p*  <  0.001; **** *p*  <  0.0001.

## 3. Results

### 3.1. Mediterranean-like Diet Reduces the Infarct Volume Following tMCAO

**Figure 2:**
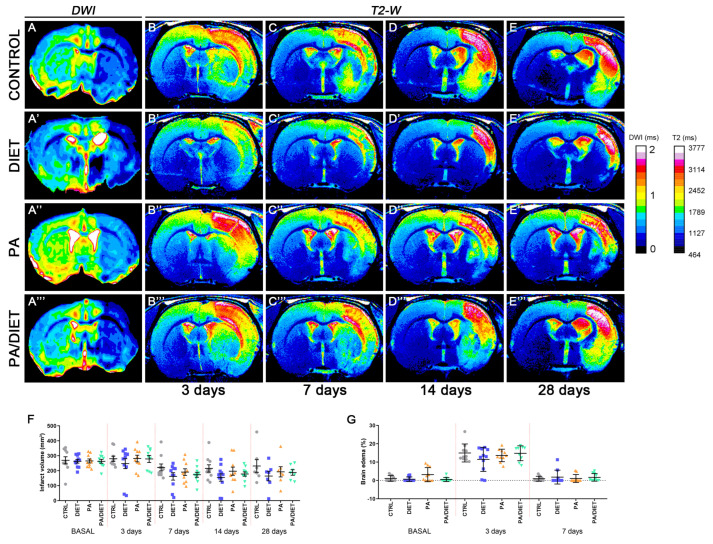
Infarct size and edema development were assessed by means of magnetic resonance imaging (MRI) (**A**–**E‴**). T2-weighted images on each row correspond to one of the 2D slices of the same animals within each group at different time points: control rats (**A**–**E**), diet group (**A′**–**E′**), PA group (**A″**–**E″**), and PA/diet group (**A‴**–**E‴**). (**F**) Time course of infarct volumes. (**G**) Time course of the edema volumes. Bars show mean ± SEM. A total of 10 rats per group from basal to 14 days, and 7 rats per group at 28 days.

We examined the effect of each condition, separately and together, on edema and the infarct volume following a stroke (Figure 2). The analysis and quantification of MR images showed that rats from all conditions had a non-significant reduction of infarct volume at the basal time after tMCAO in comparison with the control group. Although, the diet group exhibited the highest decreased trend throughout all post-stroke times, especially at 14 days post-ischemia (*p* = 0.1717) (Figure 2A–E‴,F). We also observed similar trends when assessing edema development (Figure 2G). Unexpectedly, no positive synergistic effects on infarct volume were observed for the PA/diet group. Together, these results revealed that only the Mediterranean-like diet seemed to have a positive impact on the evolution of the ischemic damage.

### 3.2. Mediterranean-like Diet Attenuates the Systemic Inflammatory Response and Reduces Oxidative DNA Damage Following tMCAO

**Figure 3:**
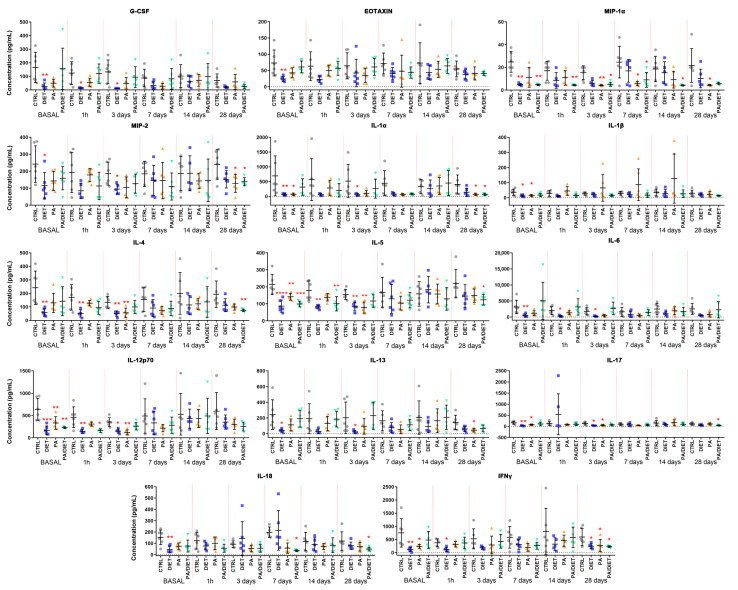
Time course of plasma concentrations of fourteen cytokines/chemokines: granulocyte colony-stimulating factor (G-CSF); eotaxin; macrophage inflammatory protein (MIP)-1α; MIP-2; interleukin (IL)-1α; IL-1β; IL-4; IL-5; IL-6; IL-12p70; IL-13; IL-17; IL-18; and interferon-gamma (IFNγ) Comparisons were made at each time point vs. control and represented in the figure if significant. * < 0.05, ** < 0.01, *** < 0.001, **** <  0.0001. *p* values are shown in Table S1. Bars show mean ± SEM. A total of 6 rats per group.

To determine the effects of either exercise or diet on the inflammatory response, pre- and post-stroke plasma samples collected were analyzed using a magnetic beads-based panel (Table S1). Remarkably, the diet group exhibited the lowest cytokine/chemokine raw values in 21 out of 27 metabolites at the basal point (pre-stroke) (Table S1). From these, levels of 14 were significantly lower in the diet group compared to controls: granulocyte colony-stimulating factor (G-CSF), eotaxin, macrophage inflammatory protein (MIP)-1α, MIP-2, interleukin (IL)-1α, IL-1β, IL-4, IL-5, IL-6, IL-12p70, IL-13, IL-17, IL-18, and interferon-gamma (IFNγ) (Figure 3, Table S1). Importantly, the Mediterranean-like diet appeared to attenuate the inflammatory response until 7 days post-stroke which showed the lowest raw levels of cytokines/chemokines compared to the controls (Table S1). Indeed, data normalization against the basal values revealed that the Mediterranean-like group strikingly increased the levels of most of the inflammatory markers from 7 days post-stroke (Figure S1, Table S1). In contrast, the PA group only showed 6 metabolites, all of them included in the diet group, with raw values that were significantly lower than the control ones at the basal point: IL-1α, IL-1β, IL-5, IL-12p70, IL-17, and IFNγ (Figure 3, Table S1). The normalized data revealed an acute increase of these basal values at 1 h, and then at 14 days post-injury in the PA group (Figure S1). The PA/diet group had 3 raw values significantly lower than the controls at the basal point: MIP-1α, IL-5, and IL-12p70 (Figure 3, Table S1). Similar to the PA group, the time-evolution of the inflammatory markers in the PA/diet group indicated an overall increase of basal values at 14 days post-injury which decreased at 28 days (Figure S1). Finally, it is important to note that all experimental conditions (diet, PA, and PA/diet) significantly decreased the raw levels of GM-CSF and INFγ compared to the control group at 28 days post-injury (Table S1).

**Figure 4:**
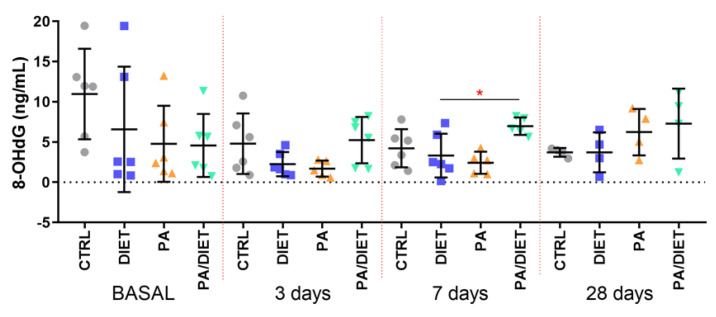
Time course of plasma 8-OHdG levels. * *p* = 0.0199. Bars show mean ± SEM. A total of 6 rats per group.

We then measured the plasma levels of 8-OHdG as a marker of oxidative stress and found that the control group exhibited the highest basal levels of 8-OhdG; although, they were not statistically significant (Figure 4). Intriguingly, the post-ischemia time points revealed that the PA/diet group had higher levels of 8-OHdG at 7 and 28 days, and that this increase was statistically significant when compared with the diet group at 7 days post-injury (Figure 4; one-way ANOVA, *p* = 0.0199); it was close to significance compared with the PA group (Figure 4; one-way ANOVA, *p* = 0.0619).

In summary, rats fed a Mediterranean-like diet exhibited the lowest pre-stroke levels of cytokines/chemokines and contained the acute inflammatory response up to 7 days pos-stroke, whereas the combination of diet and exercise seemed to block this basal anti-inflammatory response, and the PA group showed an acute increase following injury.

### 3.3. Mediterranean-like Diet Triggers Acute EPCs Mobilization

We analyzed the levels of circulating progenitor cells (CPCs, identified as CD34 positive (CD34+)), hematopoietic progenitor cells (HPCs, labeled as CD34 and CD43 positive (CD34+/CD43+), and EPCs (positive to CD34, ckit, and KDR) by flow cytometry.

**Figure 5:**
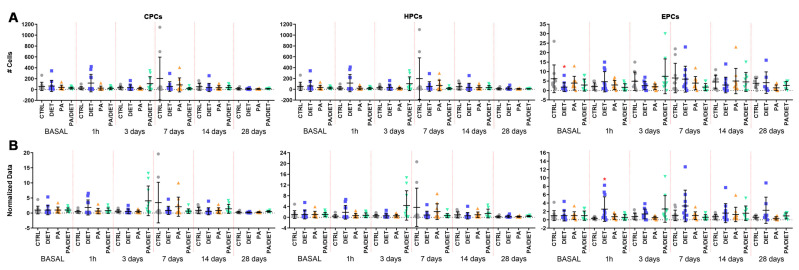
(**A**) Time course of circulating progenitor cell (CPC), hematopoietic progenitor cell (HPC), and endothelial progenitor cell (EPC) numbers from blood samples (250.000 cells counted in each one. Stats at each time point are vs. control. Bars show mean ± SEM. (**B**) Time course of normalized data from (**A**) against control values at the basal point. Stats at each time point vs. control. * < 0.05. *p* values are shown in Table S2. Bars show mean ± SEM. A total of 10 rats per group from basal to 14 days, and 7 rats per group at 28 days.

Measurements of circulating levels of CPCs and HCs showed no significant differences between groups (Figure 5A, Table S2). Regarding circulating EPCs, the diet group showed significantly lower numbers of EPCs than controls at the basal point (Kruskal–Wallis, *p* = 0.0291) (Figure 5A, Table S2). This remarkably increased at 1 h post-stroke and continued up to 28 days post-injury, as confirmed by normalized analyses against basal values showing the evolution of EPC numbers over time (one-way ANOVA, *p* = 0.0122; Figure 5B). Therefore, the diet group had the lowest value of EPCs at the basal point, although, they then exhibited a remarkable acute increase that was maintained over time.

### 3.4. Mediterranean-like Diet Improves Functional Recovery Following an Ischemic Insult

**Figure 6:**
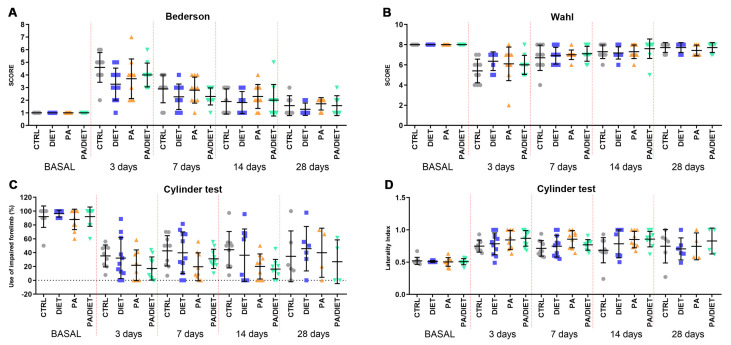
Assessment of motor and neurological behavior by the Bederson test (**A**), the Wahl test (**B**), and the cylinder test, both asymmetry (**C**) and laterality (**D**). Stats at each time point are vs. control. Bars show mean ± SEM. 10 rats per each group from basal to 14 days, and 7 rats per each group at 28 days.

Afterward, we analyzed the recovery of animals undergoing tMCAO by using different functional tests. Rats from the diet group showed the lowest values in the Bederson scale throughout all the post-injury times, especially at 3 days post-stroke, where the difference was close to statistical significance (one-way ANOVA, *p* = 0.0544) (Figure 6A). However, both the PA and PA/diet groups exhibited similar trends to the controls (Figure 6A). Likewise, the diet group showed the lowest non-significant reduction in the Wahl scale at 3 days post-injury (Kruskal–Wallis, *p* = 0.1503) (Figure 6B), but eventually, the evolution up to 28 days following injury was similar between groups (Figure 6B). Strikingly, the cylinder test revealed that the PA and PA/diet groups had the worst scores in both the use of the impaired forelimb and laterality index from 3 to 14 days following injury; although, these were not statistically significant (Figure 6C,D). The diet group mimicked control scores until 28 days post-stroke (Figure 6C,D). Overall, the diet group exhibited a better functional recovery following stroke.

### 3.5. Each Condition Acts Differently on Molecular Mechanisms Underlying Functional Improvement Following tMCAO

**Figure 7:**
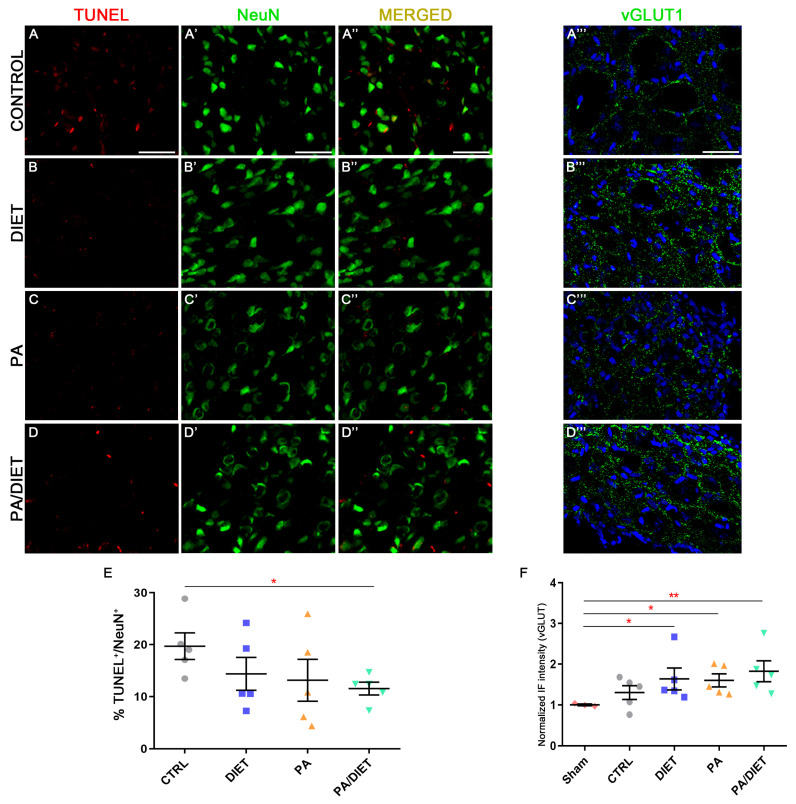
Representative images of TUNEL (**A**–**D**), NeuN (**A′**–**D′**), and vGLUT1 (**A‴**–**D‴**) staining at the peri-infarct area in the sensorimotor cortex of the control group (**A**–**A‴**); diet group (**B**–**B‴**); PA group (**C**–**C‴**); and PA/diet group (**D**–**D‴**). (**E**) Quantification of the percentage (%) of NeuN+ cells with TUNEL labeling, * *p* = 0.0149. (**F**) Quantification of vGLUT1 immunoreactivity, * *p* = 0.0153 (diet group), * *p* = 0.0376 (PA group), ** *p* = 0.003 (PA/diet group). Scale bars: 100 μm. Bars show mean ± SEM. A total of 3 sham rats and 5 rats per remaining group were used in all experiments, with 250 cells quantified per animal.

We observed higher vGLUT1 immunoreactivity in the diet, PA, and PA/diet groups than both the control and sham groups in the peri-infarct tissue at cortical layers V/VI after 28 days post-injury; although, this was statistically significant only in those against the sham group (Kruskal–Wallis, diet: *p* = 0.0153; PA: *p* = 0.0376; PA/diet: *p* = 0.0030) (Figure 7D–D‴,F). Moreover, this agreed with the analysis of TUNEL labeling in NeuN+ cells at cortical layers V/VI, where less apoptotic neuronal death was detected in all experimental conditions compared to controls; although, this difference was only significant in the PA/diet group (one-way ANOVA, *p* = 0.0149) (Figure 7A–C‴,E). Altogether, all experimental conditions were able to increase the numbers of cortical glutamatergic pyramidal neurons near the lesion, and this was especially remarkable in the PA/diet group.

**Figure 8:**
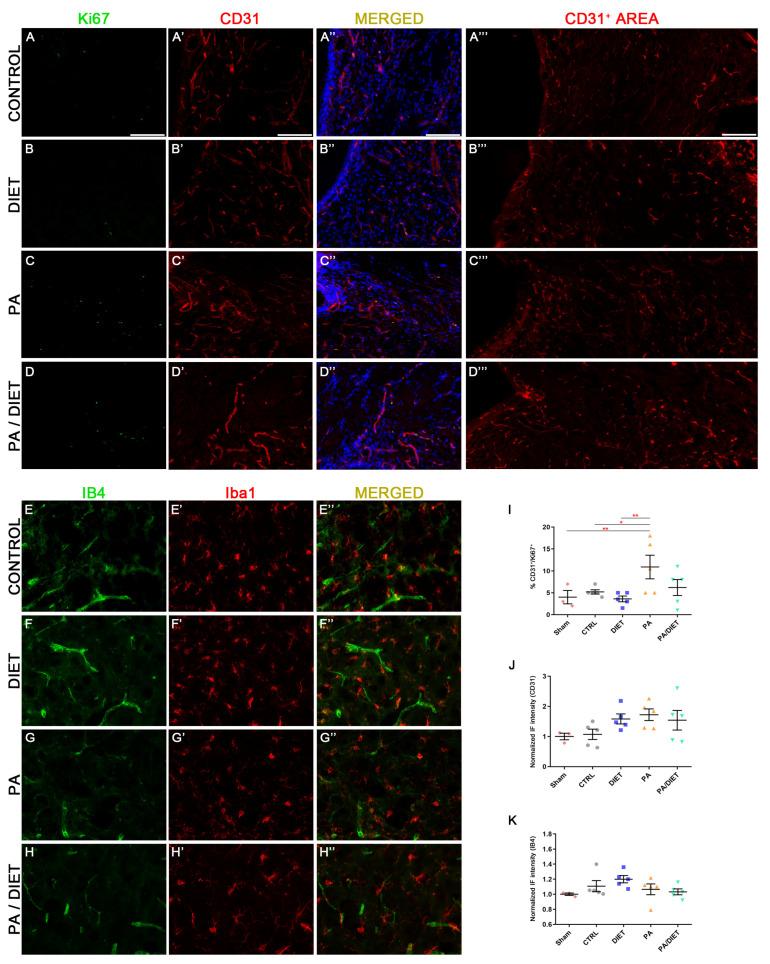
Representative images of Ki67 (**A**–**D**), CD31 (**A′**–**D′**), and CD31+ area (**A‴**–**D‴**) staining at the subventricular zone (SVZ) of the control group (**A**–**A‴**); diet group (**B**–**B‴**); PA group (**C**–**C‴**); and PA/diet group (**D**–**D‴**). Representative images of IB4 (**E**–**H**) and Iba1 (**E′**–**H′**) staining at the peri-infarct area in the sensorimotor cortex of the control group (**E**–**E″**); diet group (**F**–**F″**); PA group (**G**–**G″**); and PA/diet group (**H**–**H″**). (**I**) Quantification of the percentage (%) of CD31+ cells with Ki67 labeling from the total number of CD31+ cells, ** *p* = 0.0071 (PA vs. sham), * *p* = 0.0446 (PA vs. control), ** *p* = 0.0018 (PA vs. diet). (**J**) Quantification of CD31+ area immunoreactivity. (**K**) Quantification of IB4 immunoreactivity. Scale bars: 400 μm. Bars show mean ± SEM. A total of 3 sham rats and 5 rats per remaining group were used in all experiments, with 250 cells quantified per animal.

Our results showed that the PA group remarkably increased the percentage of CD31+/Ki67+ vessels at the subventricular zone (SVZ), not only compared to sham and control groups (one-way ANOVA, *p* = 0.0071 and *p* = 0.0446, respectively) but also compared to the diet group at 28 days post-stroke (one-way ANOVA, *p* = 0.0018) (Figure 8A–E″,J). Furthermore, these results agreed with the higher CD31+ areas seen in the diet, PA, and PA/diet groups compared to controls (Figure 8A‴–D‴,K). We also observed the pattern of expression of the IB4 staining in the peri-infarct area 28 days following stroke. The diet group exhibited the highest increase in IB4 staining but it was not statistically significant (Figure 8F,I). Our results may suggest the existence of more vascular repair in the PA group than the others after tMCAO.

**Figure 9:**
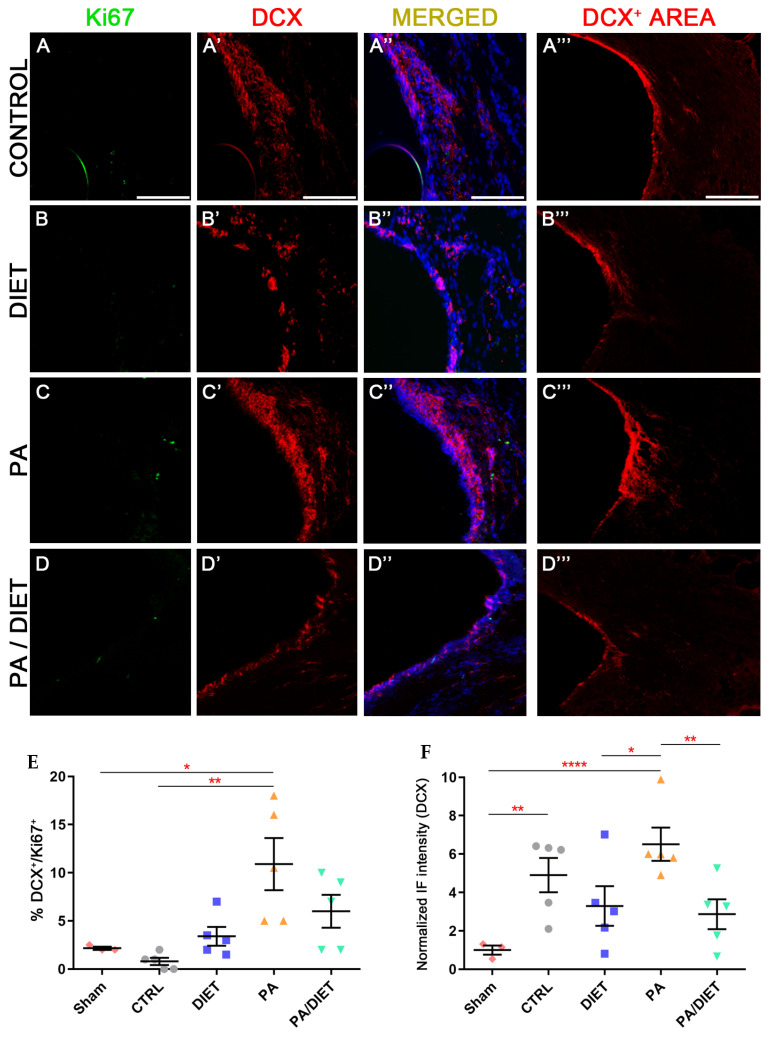
Representative images of Ki67 (**A**–**D**), DCX (**A′**–**D′**), and DCX+ area (**A‴**–**D‴**) staining at the SVZ of the control group (**A**–**A‴**); diet group (**B**–**B‴**); PA group (**C**–**C‴**); and PA/diet group (**D**–**D‴**). (**E**) Quantification of the percentage (%) of DCX+ cells with Ki67 labeling from the total number of DCX+ cells, * *p* = 0.0468 (PA vs. sham), ** *p* = 0.0013 (PA vs. control). (**F**) Quantification of DCX+ area immunoreactivity, ** *p* = 0.0055 (control vs. sham), **** *p* < 0.0001 (PA vs. sham), * *p* = 0.0239 (PA vs. diet), ** *p* = 0.0083 (PA vs. PA/diet). Scale bars: 100 μm. Bars show mean ± SEM. A total of 3 sham rats and 5 rats per remaining group were used in all experiments, with 250 cells quantified per animal.

Regarding neurogenesis, only the PA group showed statistically higher levels of neurogenesis (DCX+/Ki67+ cells) than the sham and control groups at the SVZ (Kruskal-Wallis, *p* = 0.0468 and *p* = 0.0013, respectively) (Figure 9A–D‴,E). Regarding the DCX+ area, the PA group exhibited remarkably higher labeling than the rest of the conditions (one-way ANOVA, vs. sham: *p* < 0.0001, vs. diet: *p* = 0.0239, vs. PA/diet: *p* = 0.0083) (Figure 9A‴–D‴,F). The control group also had a significantly higher DCX+ area than the sham animals at the SVZ (one-way ANOVA, *p* = 0.0055) (Figure 9A‴,F). 

In summary, rats performing physical exercise showed more angiogenesis and neurogenesis than the other conditions at 28 days post-injury. 

**Figure 10:**
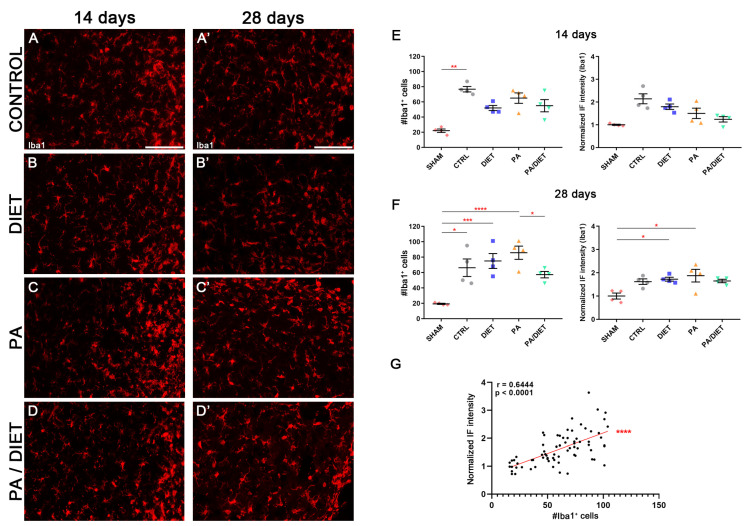
Late microglia activation in either exercise or Mediterranean-like diet or combined groups following injury. Representative images of Iba1 staining at 14 days (**A**–**D**) and 28 days (**A′**–**D′**) following ischemia in the peri-infarct area of control, diet, PA, and PA/diet groups. (**E**) Quantifications of Iba1 immunoreactivity and the number of Iba1+ cells at 14 days after injury, ** *p* = 0.0096. (**F**) Quantifications of Iba1 immunoreactivity and the number of Iba1+ cells at 28 days after injury, Iba1+ cells: * *p* = 0.0114 (control vs. sham), *** *p* = 0.0005 (diet vs. sham), **** *p* < 0.0001 (PA vs. sham), * *p* = 0.0404 (PA vs. PA/diet); Iba1 immunoreactivity: * *p* = 0.0359 (diet vs. sham), * *p* = 0.012 (PA vs. sham). (**G**) Positive correlation for Iba1 immunofluorescence (IF) signal and the number of Iba1+ cells. Scale bars: 100 μm. Bars show mean ± SEM. A total of 4 rats were used per group.

We used the Iba1 antibody, the microglia-specific marker, in the cortical peri-infarct area at two different time points after tMCAO: 14 and 28 days. After 14 days post-stroke, the control group showed higher non-significant levels of Iba1+ compared to the sham group (Kruskal–Wallis, *p* = 0.0608) and the other conditions (Figure 10A–D,F). Interestingly, this was accompanied by a significant increase in the number of Iba1+ cells from the control group compared to sham animals (Kruskal–Wallis, *p* = 0.0096) (Figure 10A–D,F). At 28 days post-injury, the immunohistochemical signal in the control group was reduced, and the diet group showed almost the same value as at 14 days, but it was statistically significant vs. the sham group (one-way ANOVA, *p* = 0.0359) (Figure 10A′–D′,G). Likewise, the PA group exhibited the highest increase which was significant vs. sham animals (one-way ANOVA, *p* = 0.012) (Figure 10C′,G). Moreover, the presence of Iba1+ cells was increased in both diet and PA groups compared to sham animals (Kruskal–Wallis, *p* = 0.0005 and *p* < 0.0001, respectively), whereas the control group showed a slight, but still significant, reduction vs. the sham group (Kruskal–Wallis, *p* = 0.0114); the PA/diet group remain unchanged (Figure 10G). Importantly, there was a positive correlation for the Iba1+ immunofluorescence signal with the numbers of Iba1+ cells surrounding the peri-infarct cortical area (Spearman test, r = 0.6444, *p* < 0.0001) (Figure 10G). Together, these results suggested that increases in the Iba1 signal are due to a higher number of Iba1+ cells, rather than an enlargement of the cell body and/or increased expression of Iba1. 

## 4. Discussion

In this study, we provided new data showing that a Mediterranean-like diet supplemented with HT may act as a pre-conditioning factor that protects against acute damage following a tMCAO injury, and this beneficial effect is attenuated when these animals also perform physical activity. This represents the first in vivo demonstration that an enriched Mediterranean diet attenuates the acute systemic inflammatory response, and this leads to a reduction in the infarct and edema volumes as well as improves functional recovery at the acute phase following ischemic injury. Our data suggest that this occurs by reducing the basal levels (pre-stroke) of cytokines/chemokines which slows down the acute and harmful stroke-induced immune response up to 7 days post-injury, and the post-stroke consumption of an enriched Mediterranean diet has no apparent effects. Here we discuss the implications and possible mechanisms underlying this enriched diet-mediated protection after tMCAO.

Our findings revealed low basal levels of circulating cytokines/chemokines and a delayed immune response in rats on a pre-emptive enriched Mediterranean-like diet post-ischemia. The spleen, which is crucial in post-damage adaptive immune responses, including ischemia [60,61], appears to be positively influenced by this diet, as supported by previous studies [62,63]. Interestingly, previous studies showed that either the pre-tMCAO removal of the spleen or its post-tMCAO irradiation results in lower infarct volumes and white blood cell counts [64,65,66]. Therefore, we hypothesize that such low basal levels of circulating proinflammatory cytokines/chemokines in animals fed a Mediterranean-like diet plus HT are due to a direct impact on spleen function. Likewise, this gradual and delayed activation of the immune cascade is maintained during the acute phase (from 1 h to 3 days) which likely reduces the infarct volume. Regarding exercise, rats of the PA group also exhibited lower levels of some circulating cytokines/chemokines compared to the control group, but it is important to note that the PA had a lower impact on inflammatory response than the Mediterranean-like diet. What is more striking is that the combination of both interventions did not act synergistically to ameliorate inflammatory response. Even so, these observations are preliminary and should be further investigated to understand the complex interactions among diet, exercise, and inflammatory responses. It is important to note that the crosstalk between oxidative stress and inflammation has a major role after stroke [67]. Here, we found a surprising result when the PA/diet group showed the highest levels of 8-OHdG in plasma following tMCAO. However, recent work showed that a dose of HT of 300 mg/kg/day in animals undergoing physical exercise appears to trigger harmful effects by switching the role of HT from anti- to pro-oxidant [68], as previously suggested in vitro [69]. Therefore, our results support previous ones and point to a pro-oxidant effect of the 200 mg/kg/day dose of HT when combined with exercise, which may negatively impact the immune response following ischemic damage. Further studies will be needed to fully elucidate the mechanisms of action for each process.

Most of these circulating cytokines/chemokines target microglia and astrocytes which are pivotal players in the immune response following ischemia, being both activated within minutes after damage and having a great impact on neurotoxicity and infarct development. Although the harmful impact of astrocytes is generally accepted, there is controversy about the exact role of microglia following ischemic injury [70,71,72,73]. Remarkably, it has been shown that microglia and newly recruited macrophages keep an anti-inflammatory state, M2, at initial steps following damage that it is gradually changed to a pro-inflammatory state, M1 [74]. Based on our data, we propose that the diet-mediated low levels of pro-inflammatory cytokines/chemokines may extend this M2 state in microglia during the acute phase after ischemia which would attenuate the cerebral inflammation and, hence, reduce infarct volume. Furthermore, the number of microglia rapidly increased during the first 10 days after the insult [75]. Although we did not perform analysis at 10 days post-injury, we did observe that the number of Iba1+ cells decreased in the control group from 14 to 28 days post-injury, and this was contrary to the diet group where we found more Iba1+ cells at 28 days than at 14 days post-injury with no effect of re-starting the diet at 7 days post-injury, which is consistent with previous work at 35 days post-ischemia [31]. This agrees with the increasing levels of cytokines/chemokines seen in the diet group from 7 days post-injury and may explain this late increase in Iba1+ cells. Furthermore, the PA group exhibited a temporal increase in cytokines/chemokines at 14 days compared to 7 days post-stroke, which could explain the increased numbers of Iba1+ cells at 28 days as acute bouts of exercise transiently increase inflammation [76]. Therefore, it is plausible that basal/acute low levels of pro-inflammatory cytokines/chemokines promoted by diet/HT could slow down the activation and recruitment of microglia until the sub-chronic/chronic phase of ischemia, and that the restoration of physical exercise following injury may cause an acute increase in circulating cytokines/chemokines that eventually activates microglia. 

Vasogenic edema is the main component of brain edema, and it is caused by the disruption of endothelial tight junctions in the BBB due to inflammatory molecules and oxidative stress following brain injuries [77]. Specifically, glial and peripheral immune cells, as well as their released cytokines/chemokines, have a key role in the pathophysiology of vasogenic edema [77]. Our results from flow cytometry analysis revealed an acute increase in EPC levels only in the diet group following injury. This is a remarkable result given that these animals had the lowest level of EPCs at the basal time. Similarly, both CD34+ and HPC numbers were the highest in the diet group from basal to 3 days post-injury. Interestingly, several works assessed the effect of a Mediterranean diet on CD34+ and EPC numbers following different injuries, but not ischemic damage [38,78,79], overall, seeing an increase in EPC numbers due to the diet. Recently, an in vitro study reported that low concentrations of HT can stimulate the migration of endothelial cells [80]. Moreover, previous clinical studies have shown that CD34+ BMPCs, including EPCs, are related to a good functional recovery and outcome following a stroke [57,58,59], being consistent with the results of our study. Hence, it is conceivable that either diet or HT or both acts on CD34+ progenitor cells, primarily EPCs, allowing them to reach the site of injury. This is due to the high concentration of angiogenic factors and inflammatory cytokines, from which they paracrinally release different factors promoting angiogenesis and recruiting EPCs, which either restore the endothelium or form new vessels guided [81]. Furthermore, immunohistochemical analysis showed a higher CD31+ area but slightly lower numbers of proliferating CD31+ cells in the SVZ of the diet group, besides an increase in IB4 labeling in the peri-infarct area, compared to controls at 28 days post-injury. Overall, these results may suggest that either low neuroinflammation or increased acute levels of CD34+ BMPCs/EPCs, or both, protects BBB-forming endothelial cells and reduces cerebral edema. Given that animals fed an enriched Mediterranean-like diet displayed a larger CD31+ area and IB4 staining, but few proliferating CD31+ cells, at 28 days post-injury, it could be possible that EPCs exert a protective role rather than promoting angiogenesis. The exercise group did not show important changes in CD34+ BMPC numbers, however, it did show a remarkable increase in proliferating CD31+ cells at 28 days post-injury. Previous studies addressed the role of exercise in angiogenesis with positive results [11,82], and therefore, we speculate that animals fed a Western-like diet that underwent physical activity showed higher angiogenesis by incrementing CD31+ cell proliferation. Future studies will be needed to decipher the exact mechanism of cerebral vasculature underlying both Mediterranean diet/HT and exercise-positive outcomes.

Several studies highlighted the ability of polyphenols to trigger anti-apoptotic and antioxidant mechanisms [19,20,21,22,23,24,25]. Importantly, HT is a well-known minor component found in extra-virgin olive oil that has an important physiological effect following ischemic damage such as neuroprotection [26,27,28,29,30,31,83]. Likewise, physical exercise has been related to higher neuronal survival following injury [84,85,86,87]. Cortical pyramidal neurons at layers V/VI are the main players not only for motor functions but also for integrating sensory inputs [88,89]. As glutamatergic neurons, vesicular glutamate transporter 1 (vGLUT1) plays a vital role in glutamatergic transmission, and therefore, this has been suggested as beneficial in the chronic phase following a stroke [90,91]. Here, we reported that either diet or PA or PA/diet led to a reduced neuronal death in the cortex which agreed with a higher vGLUT1 intensity at 28 days post-injury. Based on the above previous works, we speculate that such a reduction in TUNEL labeling can be related to either the HT supplement present in the Mediterranean-like diet, exercise, or both. Interestingly, the area occupied by DCX, a marker for migrating immature neurons, was smaller in both diet and PA/diet groups than in control animals at 28 days post-injury, which could suggest that those animals fed on the enriched diet did not need to replace death neurons as much as control rats. Interestingly, a Mediterranean-like diet supplied with HT slightly maintained a high rate of proliferating neurons at 28 days post-injury compared to controls, and this trend appeared to increase in the PA/diet, which may indicate a more remarkable influence of exercise in promoting neuron proliferation. Indeed, the PA group exhibited a striking rate of neuron proliferation almost 10 times higher than the control group at 28 days post-injury, which agreed with the proliferating rate seen in CD31+ cells and may explain the highest DCX+ area in the PA group. Overall, while our results suggest that HT in a Mediterranean diet might enhance neuroprotection post-ischemia, it is important to note that these are preliminary observations. The direct impact of HT on neuroprotective mechanisms remains a subject for further investigation. This speculation is based on our current findings but should be substantiated with more focused research in the future.

Regarding functional outcomes, the consumption of a Mediterranean-like diet enriched with HT led to a clearer improvement in functional recovery at short times (3 and 7 days post-injury). The pattern of effects on neuroinflammation, infarct volume, and cellular mechanisms may support the hypothesis that an enriched Mediterranean diet allows faster behavioral recovery through a combination of these three aspects rather than one alone. In this regard, our study agrees with previous studies in that either pre-injury olive oil compounds [28,29] or post-injury HT administration [30,31] shows less infarct volume and better functional recovery in rodent models of ischemic damage. The other conditions of a PA and PA/diet also showed a better trend than controls in behavioral tests at acute times post-injury (3 days), likely by increasing cellular proliferation. However, it is also important to note that all conditions eventually achieved similar levels of functional recovery at 28 days post-injury.

A limitation of this study is the use of only male rats. Several animal studies reported gender-based differences in stroke outcome, with males showing higher infarct volumes and mortality than their female counterparts [92,93]. Although gender-based differences were not one of our aims in this study, we decided to use male rats to study the impact of the Mediterranean-like diet under a background with the maximum stroke-induced damage. Importantly, males showed higher levels of proinflammatory cytokines after stroke [93], and given the main goal of this work, we considered them the most appropriate gender to carry out our experiments.

## 5. Conclusions

In conclusion, our study confirms our initial hypothesis that an HT-enriched Mediterranean diet reduces pre-ischemic cytokines/chemokines and mitigates acute immune responses, aligning with our objective to explore dietary impacts on stroke recovery. This provides novel evidence of how such a diet modulates immune responses, contributing to quicker functional recovery post-ischemia. Unexpectedly, physical exercise exerts a dual role: it is beneficial in animals fed on a Western-like diet, while it reduces some of the beneficial outcomes promoted by a Mediterranean-like diet. Future studies will be required to determine the exact cellular mechanisms underlying the beneficial effect of either HT or the Mediterranean diet or both following ischemic damage.

## Data Availability

Data is contained within the article and Supplementary Material.

## Supplementary Materials

The following supporting information can be downloaded at: https://www.mdpi.com/article/10.3390/antiox13020138/s1, Figure S1: Time course of normalized data, against control values at basal, from concentrations of the twenty-seven cytokines/chemokines: granulocyte colony-stimulating factor (G-CSF); eotaxin; macrophage inflammatory protein (MIP)-1α; MIP-2; interleukin (IL)-1α; IL-1β; IL-4; IL-5; IL-6; IL-12p70; IL-13; IL-17; IL-18; interferon gamma (IFNγ); regulated on activation, normal T cell expressed and secreted (RANTES); tumor necrosis factor-alpha (TNFα); vascular endothelial growth factor (VEGF); epidermal growth factor (EGF); fractalkine; granulocyte-macrophage colony-stimulating factor (GM-CSF); GRO/KC; IL-2; IL-10; interferon gamma-induced protein 10 (IP-10); leptin; LPS-induced CXC (LIX); and monocyte chemoattractant protein-1 (MCP-1). Comparisons were made at each time point vs. control and represented in the figure if significant. * < 0.05, ** < 0.01, *** < 0.001. *p* values are shown in Table S1. Bars show mean ± SEM. 6 rats per each group. Table S1: Raw and normalized data from the Milliplex assay; and Table S2: Raw and normalized data from the flow cytometry analysis of hematopoietic lineages.
