# Supplementary material for: Neuroprotection Afforded by an Enriched Mediterranean-like Diet Is Modified by Exercise in a Rat Male Model of Cerebral Ischemia
Source: Antioxidants (Basel). 2024 Jan 23;13(2):138. doi: 10.3390/antiox13020138 (PMC10885962; doi:10.3390/antiox13020138)

Supplementary information

Neuroprotection afforded by an enriched Mediterranean-like diet is modified by exercise in a rat model of cerebral ischemia

Supplementary figure with legend

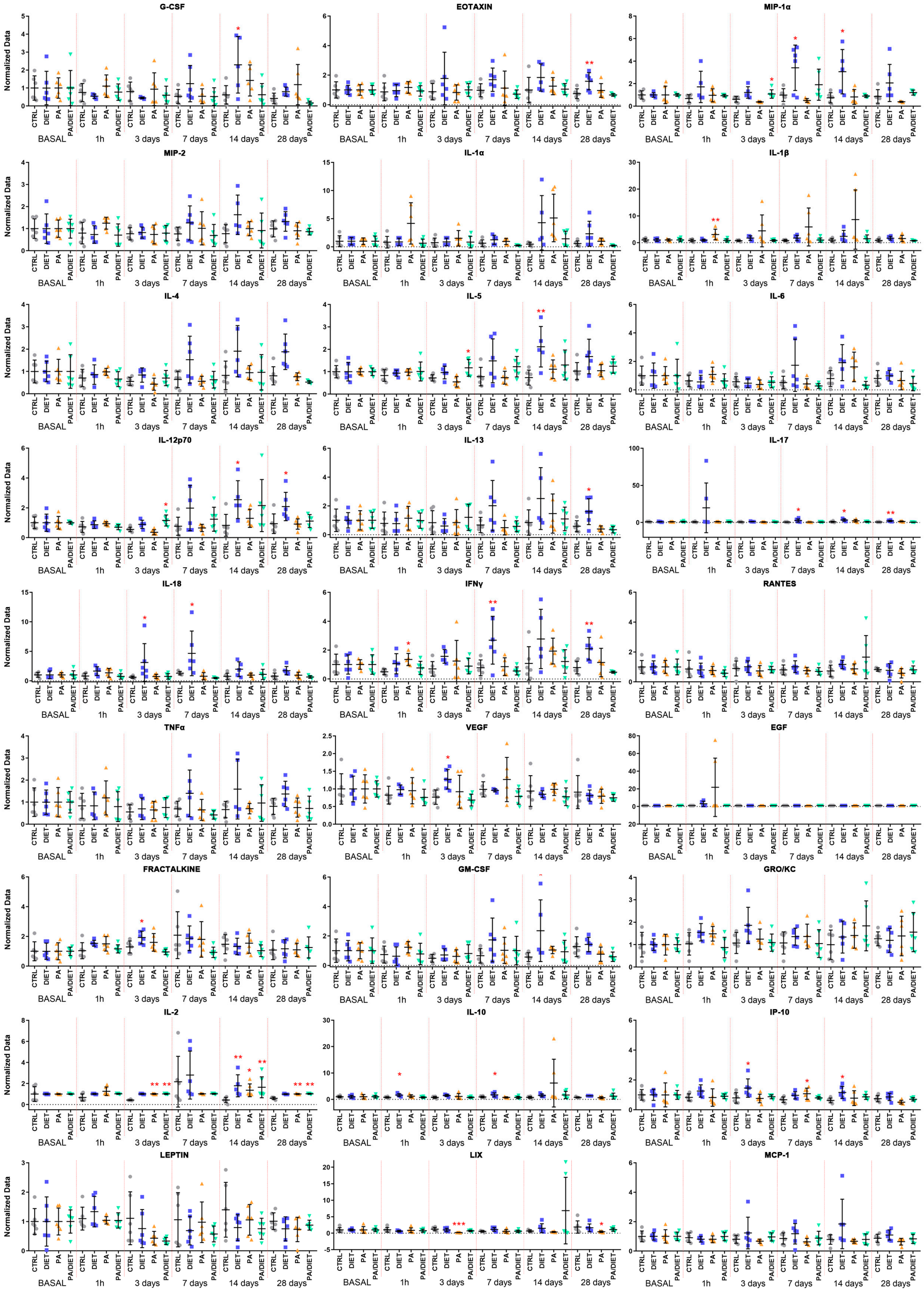

Supplement: Supplementary file 1 [file antioxidants-13-00138-s001.zip › Figure S1.pdf]
